# Development and validation of a preoperative CT‑based radiomics nomogram to differentiate tuberculosis granulomas from lung adenocarcinomas: an external validation study

**DOI:** 10.1186/s12885-024-12422-3

**Published:** 2024-06-01

**Authors:** Liping Yang, Zhiyun Jiang, Jinlong Tong, Nan Li, Qing Dong, Kezheng Wang

**Affiliations:** 1 Department of PET-CT, Harbin Medical University Cancer Hospital, Harbin, China; 2 Medical Imaging Department, Harbin Medical University Cancer Hospital, Harbin, China; 3 Medical Imaging Department, The Fourth Affiliated Hospital of Harbin Medical University, Harbin, China; 4 Department of Pathology, The Fourth Affiliated Hospital of Harbin Medical University, Harbin, China; 5 Department of Thoracic Surgery, The Fourth Affiliated Hospital of Harbin Medical University, Harbin, China

**Keywords:** Lung adenocarcinoma, Tuberculosis granuloma, Radiomics, Nomogram, Computed tomography

## Abstract

**Background:**

An accurate and non-invasive approach is urgently needed to distinguish tuberculosis granulomas from lung adenocarcinomas. This study aimed to develop and validate a nomogram based on contrast enhanced-compute tomography (CE-CT) to preoperatively differentiate tuberculosis granuloma from lung adenocarcinoma appearing as solitary pulmonary solid nodules (SPSN).

**Methods:**

This retrospective study analyzed 143 patients with lung adenocarcinoma (mean age: 62.4 ± 6.5 years; 54.5% female) and 137 patients with tuberculosis granulomas (mean age: 54.7 ± 8.2 years; 29.2% female) from two centers between March 2015 and June 2020. The training and internal validation cohorts included 161 and 69 patients (7:3 ratio) from center No.1, respectively. The external testing cohort included 50 patients from center No.2. Clinical factors and conventional radiological characteristics were analyzed to build independent predictors. Radiomics features were extracted from each CT-volume of interest (VOI). Feature selection was performed using univariate and multivariate logistic regression analysis, as well as the least absolute shrinkage and selection operator (LASSO) method. A clinical model was constructed with clinical factors and radiological findings. Individualized radiomics nomograms incorporating clinical data and radiomics signature were established to validate the clinical usefulness. The diagnostic performance was assessed using the receiver operating characteristic (ROC) curve analysis with the area under the receiver operating characteristic curve (AUC).

**Results:**

One clinical factor (CA125), one radiological characteristic (enhanced-CT value) and nine radiomics features were found to be independent predictors, which were used to establish the radiomics nomogram. The nomogram demonstrated better diagnostic efficacy than any single model, with respective AUC, accuracy, sensitivity, and specificity of 0.903, 0.857, 0.901, and 0.807 in the training cohort; 0.933, 0.884, 0.893, and 0.892 in the internal validation cohort; 0.914, 0.800, 0.937, and 0.735 in the external test cohort. The calibration curve showed a good agreement between prediction probability and actual clinical findings.

**Conclusion:**

The nomogram incorporating clinical factors, radiological characteristics and radiomics signature provides additional value in distinguishing tuberculosis granuloma from lung adenocarcinoma in patients with a SPSN, potentially serving as a robust diagnostic strategy in clinical practice.

**Supplementary Information:**

The online version contains supplementary material available at 10.1186/s12885-024-12422-3.

## Introduction

The differentiation between peripheral lung cancer and tuberculosis granuloma is still a challenging issue [1]. Lung tuberculosis manifested as nodular or mass is easily misdiagnosed as peripheral lung cancer [2]. However, the treatment options and clinical prognosis between lung cancer and tuberculosis are completely distinct. Radical surgical resection is the first choice for the former, while the latter tends to be treated with anti-tuberculosis drugs [3]. Misdiagnosis will cause unnecessary treatment and financial burden, especially when the diagnosis of lung adenocarcinoma is delayed. Patients might lose the best chance for treatment, leading to uncontrollable tumor progression and poorer prognosis [4]. Therefore, finding an accurate and non-invasive approach to differentiate lung cancer from tuberculosis is of great significance, which undoubtedly has become an important topic for radiologists and clinicians.

The contrast enhanced-compute tomography (CE-CT) is widely applied to distinguish different lung diseases mainly dependent on morphological features, such as speculation, contour, and border definition, which have shown improved diagnostic performance [5]. Unfortunately, nodular/mass pulmonary tuberculosis granuloma usually appears as round or irregular mass shadows in CT images, and its edges may be lobed or manifested as a spicule sign [6]. As a result of non-specific clinical and radiological manifestations, diagnostic accuracy is closely related to the radiologists’ knowledge level and working experience. Despite the rapid development of CT-guided biopsy technology, a series of adverse complications severely restrict the wide application of this operation for pathological diagnosis [7].

Radiomics is a promising and non-invasive method that can extract automatic high-throughput quantitative features from images [8]. It captures relationships between image voxels that the naked eye of physicians may not perceive-even experienced radiologists, which can contribute to the diagnostic and predictive accuracy of the disease. Therefore, this study was aimed to develop and validate a radiomics nomogram based on preoperative CE-CT images to differentiate tuberculosis granuloma from lung adenocarcinoma presenting as solid nodules or masses.

## Materials and methods

### Study population

**Fig. 1:**
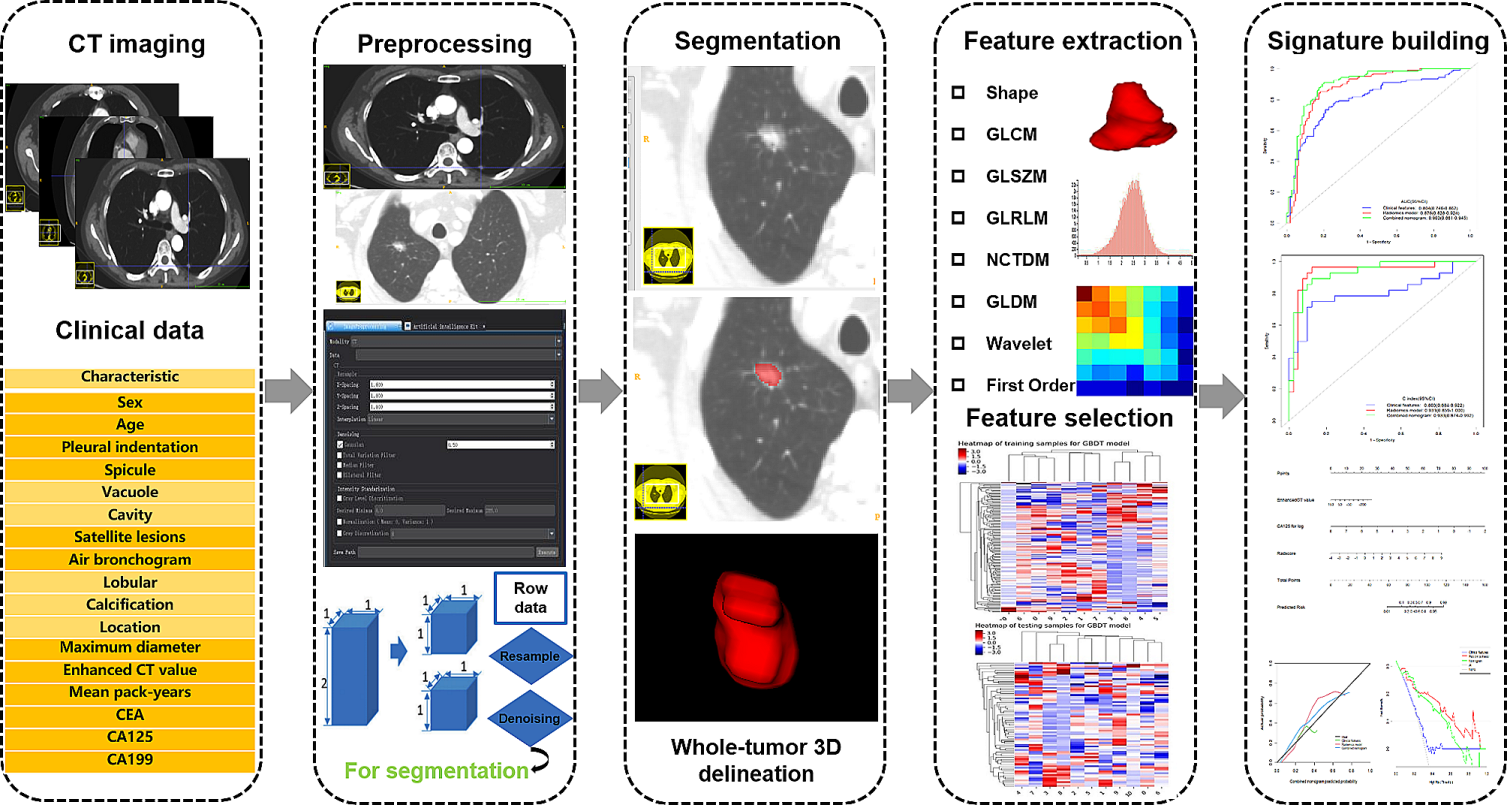
The flow diagram of this study

The retrospective research protocol was reviewed, approved, and overseen by the institutional review board of Harbin Medical University Cancer Hospital and The Fourth Affiliated Hospital of Harbin Medical University, and the need for written informed consent was waived. The enrollment flowchart of this study was displayed in Fig. 1. Detailed inclusion criteria were listed as follows: (a) solitary and solid nodules or masses, which may contain cavities or vacuoles and do not exhibit a ground glass density; (b) histological diagnosis confirmed by surgical resection; and (c) available preoperative chest CT images (within 4 weeks prior to surgery). Detailed exclusion criteria were described as follows: (a) ambiguous pathological diagnosis from insufficient tissue samples; (b) subsolid pulmonary nodules, including non-solid nodules and partly solid nodules; (c) patients with a history of surgery, radiotherapy, or chemotherapy; (d) patients with a history of other malignant tumors and (e) substandard image quality, such as motion artifacts.

### Image acquisition and image analysis

All chest CT images were obtained from Discovery CT 750 HD (GE Medical Systems, USA). Scans were acquired from the thoracic inlet to the level of the bilateral adrenal glands during deep inspiration breath-hold. Detailed scanning parameters were as follows: tube voltage 100-140 kV, tube current 350-550 mA, slice thickness 3 mm, reconstruction interval 3 mm, matrix size 512 × 512, and field of view 450 mm. Non-ionic contrast media (Iohexeol, 350 mg/ml, GE, Boston, USA) was administered at a rate of 3.0-3.5 ml/s and 1.2 ml/kg to all patients, as standardized protocol. Arterial phase (AP) images were scanned around 30s post-injection of contrast media. Two experienced radiologists who both has 10-year practicing experience in chest disease diagnosis, blinded to the pathological results, independently evaluated the CT images. The details of the evaluation of subjective CT findings were displayed in Supplementary Materials (Supplementary data 1).

### Tumor segmentation and radiomics feature extraction

Three steps were adopted to preprocess the CT images before feature extraction. Firstly, all images were resampled to a uniform voxel size of 1 mm × 1 mm × 1 mm using linear interpolation to minimize the influence of different layer thicknesses. Secondly, the continuous images were converted into discrete values based on the gray-scale discretization process (bin width = 25). Finally, the Laplacian of Gaussian and wavelet image filters were used to eliminate the mixed noise in the image digitization process to obtain low- or high-frequency features. Axial CT Digital Imaging and Communications in Medicine images were applied for tumor segmentation. The tumor lesion was delineated on axial CT images using ITK-SNAP software (version 3.6.0, www.itksnap.org). Radiomics features were extracted from each CT-derived volume of interest (VOI) by applying dedicated AK software (Artificial Intelligence Kit, GE Healthcare), which complies with image biomarker standardization initiative guidelines [9]. A total of 851 radiomics features were extracted from each VOIs, and the classification of various features was listed in Supplementary Materials (Supplementary data 2).

### Radiomics feature selection and model establishment

Intra- and inter-class correlation coefficients (ICCs) were calculated to evaluate the intra- and inter-observer reproducibility. Two readers drew the volumes of interest (VOIs) on 40 randomly selected CT images (20 cases of tuberculosis granulomas and 20 cases of lung adenocarcinomas). Reader 1 repeated the segmentations two weeks later. An ICC greater than 0.80 indicated good agreement with feature extraction. The VOI segmentation for the remaining cases was performed by Reader 1. After the intra- and inter-operator agreement evaluation, radiomics features with ICC > 0.80 were selected for further analysis. Next, the feature selection was carried out by using a step-by-step selection method. Firstly, univariate logistic regression analysis was applied to select features with *P*-value < 0.05 for the subsequent analysis. Secondly, multivariate logistic regression analysis was utilized to choose features closely related to pulmonary nodules classification. Finally, the most informative features were retained using the least absolute shrinkage and selection operator (LASSO) method. LASSO regression shrinks the coefficient estimates toward zero, with the degree of shrinkage dependent on an additional parameter, alpha. To determine the optimal values for alpha, a 10-time cross-validation was used, and we chose alpha via the minimum criteria and a value of ln (alpha)= -2.1 was chosen.

### Model building and evaluation of predictive models

A clinical model for predicting pulmonary nodule classification was developed using univariable and multivariable logistic regression analysis. The following candidate predictors were considered: tumor size, location, cavity, vacuole, spicule, satellite lesions, calcification, lobulation, pleural indentation, and air bronchogram. Furthermore, a nomogram was created based on both clinical-radiological parameters and the combined radiomics signature. The diagnostic performance of the different models was evaluated in the training and testing sets by assessing sensitivity, specificity, and accuracy. The calibration curve was employed to assess the agreement between the nomogram’s prediction results and actual clinical findings. Decision curve analysis (DCA) was used to validate the clinical usefulness of the radiomics nomogram.

### Statistical analysis

Statistical analysis for the present study was conducted using the IPM Statistics program (V 2.1.0.R), R (version 3.5.1), and Python (version 3.5.6). Categorical variables were compared using either a chi-squared test or Fisher’s exact test, with *P* < 0.05 denoting a significant difference. The receiver operating characteristic (ROC) curve was constructed to evaluate the discriminative performance of each model. A two-sided *P* value < 0.001 was considered statistically significant.

## Results

### Clinical characteristics of patients

Patient demographics and CT characteristics of all patients were presented in Table 1. A total of 280 patients from the two centers between March 2015 to June 2020 were enrolled in this study. The training and internal validation cohorts included 161 and 69 patients from center No.1, respectively. The external test cohort included 50 patients from center No.2. In the univariate regression analysis, the classification of solitary pulmonary nodules had significant associations with sex, age, mean pack-years, spiculation, air bronchogram, maximum diameter, enhanced-CT value, CEA, CA125 and CA199 (both *P* < 0.001). In the multivariate regression analysis, only enhanced-CT value and CA125 were demonstrated to be independent predictors (both *P* < 0.001). The results of univariate and multivariate regression analysis were displayed in Table 2.

**Table 1:** Baseline clinical characteristics of patients

	Training cohort (*n* = 161)			Internal validation cohort (*n* = 69)			External test cohort (*n* = 50)		
	Adenocarcinoma	Granuloma		Adenocarcinoma	Granuloma		Adenocarcinoma	Granuloma	
Sex									
Male	29	66		16	20		20	11	
Female	39	27		25	8		14	5	
**Age (years)**	61.8 ± 8.7	52.3 ± 10.0		63.5 ± 7.3	51.7 ± 8.8		65.0 ± 4.2 59.8 ± 9.7		
**Mean pack-years**	26.3 (20.9, 31.9)	14.9 (10.6, 17.8)		29.5 (20.5, 43.5)	15.6 (12.1, 17.5)		23.8 10.8 (19.6, 35.9) (9.9,16.9)		
**Pleural indentation**									
Absence	3	14		2	4		9	3	
Presence	65	79		39	24		25	13	
**Spicule**									
Absence	7	36		3	10		5	6	
Presence	61	57		38	18		29	10	
**Vacuole**									
Absence	59	83		39	21		26	14	
Presence	9	10		2	7		8	2	
**Cavity**									
Absence	63	86		40	27		31	15	
Presence	5	7		1	1		3	1	
**Satellite lesions**									
Absence	68	78		40	25		34	14	
Presence	0	15		1	3		0	2	
**Air bronchogram**									
Absence	59	89		34	27		31	13	
Presence	9	4		7	1		3	3	
**Lobular**									
Absence	0	2		1	0		0	1	
Presence	68	91		40	28		34	15	
**Calcification**									
Absence	57	89		40	26		33	14	
Presence	1	4		1	2		1	2	
**Location**									
Right upper lobe	27	30		12	14		17	5	
Right middle lobe	6	3		0	1		3	1	
Right lower lobe	7	19		11	5		3	0	
Left upper lobe	15	29		11	6		6	8	
Left lower lobe	13	12		7	2		5	2	
**Maximum diameter (mm)**	30.6 (24.6, 40.9)	22.7 (18.7, 31.2)		34.1 (24.7, 52.3)	23.0 (18.7, 31.4)		21.1 (13.7, 30.2)	20.6 (14.7, 23.7)	
**Enhanced CT value (HU)**	60.7 (52.6, 76.6)	36.8 (22.0, 54.8)		62.6 (55.7, 79.4)	39.9 (19.9, 59.7)		66.1 (51.9, 80.1)	47.5 (35.0, 59.8)	
**CEA (ng/ml)**	4.4 (2.3, 15.9)	2.5 (1.47, 3.9)		5.9 (2.3, 36.8)	2.2 (1.6, 3.7)		12.4 (3.5, 66.2)	3.2 (2.1, 8.4)	
**CA125 (ng/ml)**	13.5 (9.9, 18.0)	3.4 (2.3, 7.45)		12.9 (9.8, 20.6)	6.9 (2.9, 9.7)		15.3 (9.9, 33.1)	4.7 (3.7, 7.4)	
**CA199 (ng/ml)**	10.9 (4.5, 17.4)	7.4 (3.3, 10.8)		12.1 (5.8, 17.9)	6.6 (2.2, 9.8)		13.3 (5.6, 18.9)	5.8 (2.9, 10.2)	

*Note * Continuous variables are expressed as Median (interquartile range). Otherwise, data are number of patients. CEA (carcinoembryonic antigen); CA153 (carbohydrate antigen 153); CA199 (carbohydrate antigen 199)

**Table 2:** Results of and univariate and multivariate logistic regression

	Univariate logistic regression			Multivariate logistic regression		
	OR	95% CI	*P* value	OR	95% CI	*P* value
Sex	3.495	2.021–6.042	0.000	1.542	0.484–4.911	0.464
Age (years)	1.138	1.096–1.181	0.000	1.130	1.048–1.218	0.102
Mean pack-years	1.553	1.351–1.785	0.000	1.352	1.167–1.1566	0.060
Pleural indentation	3.635	1.301–10.157	0.014	1.085	0.133–8.863	0.939
Spicule	6.072	2.877–12.813	0.000	3.975	0.808–19.557	0.090
Vacuole	0.687	0.306–1.539	0.361	0.599	0.422–1.004	0.644
Cavity	0.823	0.276–2.451	0.726	0.479	0.365–0.988	0.512
Satellite lesions	0.053	0.007 0.404	0.006	0.465	0.029–7.433	0.589
Air bronchogram	3.991	1.410-11.299	0.009	10.081	0.678–14.997	0.093
Lobular	1.815	0.162–20.302	0.628	1.003	0.997–1.451	0.885
Calcification	0.358	0.071–1.814	0.215	0.309	0.211–0.885	0.703
Location	1.050	0.249–4.422	0.947	0.966	0.704–1.113	0.472
Maximum diameter	1.056	1.031–1.081	0.000	1.022	0.971–1.075	0.412
Enhanced CT value	2.034	1.347–2.981	0.000	2.389	1.756–3.247	0.000
CEA (ng/ml)	1.246	1.112–1.395	0.000	1.033	0.902–1.184	0.637
CA125 (ng/ml)	1.319	1.223–1.422	0.000	1.312	1.134–1.518	0.000
CA199 (ng/ml)	1.073	1.033–1.114	0.000	0.993	0.967–1.021	0.628

### Intra and inter-observer reproducibility of feature extraction

The intra-observer ICC ranged from 0.815 to 0.940, and inter-observer ICCs ranged from 0.720 to 0.906. Therefore, a favorable intra- and inter-observer reproducibility of radiomics feature extraction was observed in our study.

### Feature selection and model building

**Fig. 2:**
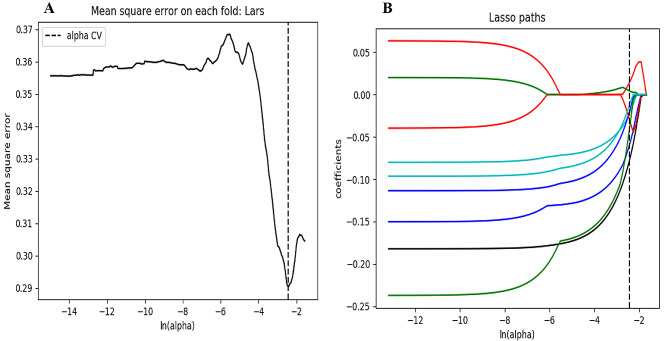
Selection of significant parameters in radiomics features in the training cohort and definition of linear predictor. **(a)** Ten time cross-validation for tuning parameter selection in the LASSO model. **(b)** LASSO

The clinical model was developed based on multivariable logistic regression, and it identified enhanced-CT value (odds ratio [OR], 2.389; 95% confidence interval [CI], 1.756–3.247; *P* < 0.001) and CA125 (OR, 1.312; 95% CI, 1.134–1.518; *P* < 0.001) as independent predictors. Initially, 850 radiomics features were extracted from each VOI of the CT image. All obtained radiomics features underwent preprocessing and standardization using the z-score approach. Subsequently, 720 radiomics features were selected based on a repeatability standard of ICC ≥ 0.80. Univariate and multivariate logistic regression analyses were further performed to reduce the dimensions of these features. Then, 9 features were selected as the most predictive subset to construct radiomics model after LASSO (Fig. 2A, B). The selected radiomics features and corresponding coefficients were listed in Table 3. The nomogram was created to differentiate tuberculosis granulomas from lung adenocarcinomas, incorporating two factors (enhanced-CT value and CA125) and the radiomics signature (namely rad-score).

**Table 3:** The selected radiomics features and corresponding coefficients

Variables	Coefficients
original_firstorder_10Percentile	-0.03942
original_glcm_MaximumProbability	20.82
original_shape_Flatness	-3.425
wavelet.HHH_glcm_MCC	2.369
wavelet.HHL_firstorder_Mean	-1.049
wavelet.HHL_firstorder_Median	-28.89
wavelet.HHL_glcm_Imc1	-30.42
wavelet.LHH_firstorder_Skewness	0.1982
wavelet.LLH_ngtdm_Complexity	0.000000852

Rad-score = 0.5014 − 0.03942*original_firstorder_10Percentile + 20.82*original_glcm_MaximumProbability-3.425*original_shape_Flatness + 2.369*wavelet.HHH_glcm_MCC-1.049*wavelet.HHL_firstorder_Mean-28.89*wavelet.HHL_firstorder_Median-30.42*wavelet.HHL_glcm_Imc1 + 0.1982*wavelet.LHH_firstorder_Skewness + 0.0000008524*wavelet.LLH_ngtdm_Complexity.

### Performance of three prediction models

**Fig. 3:**
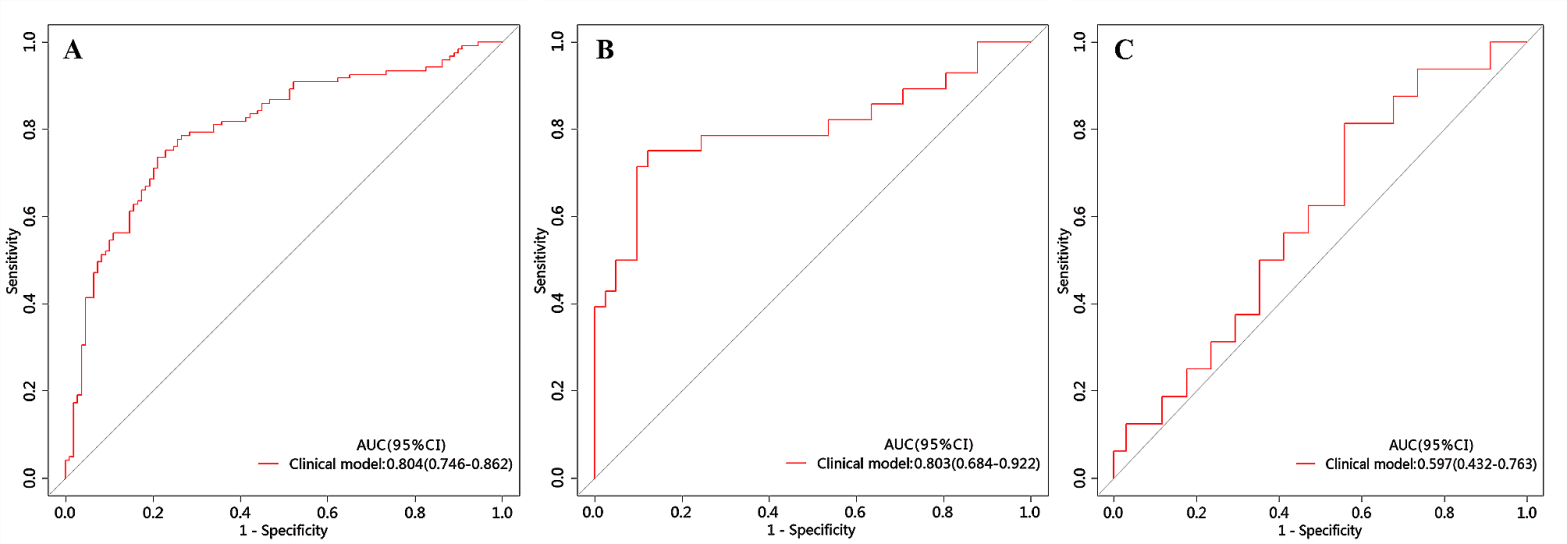
ROC of the clinical model in the training, internal validation and external testing cohorts

**Fig. 4:**
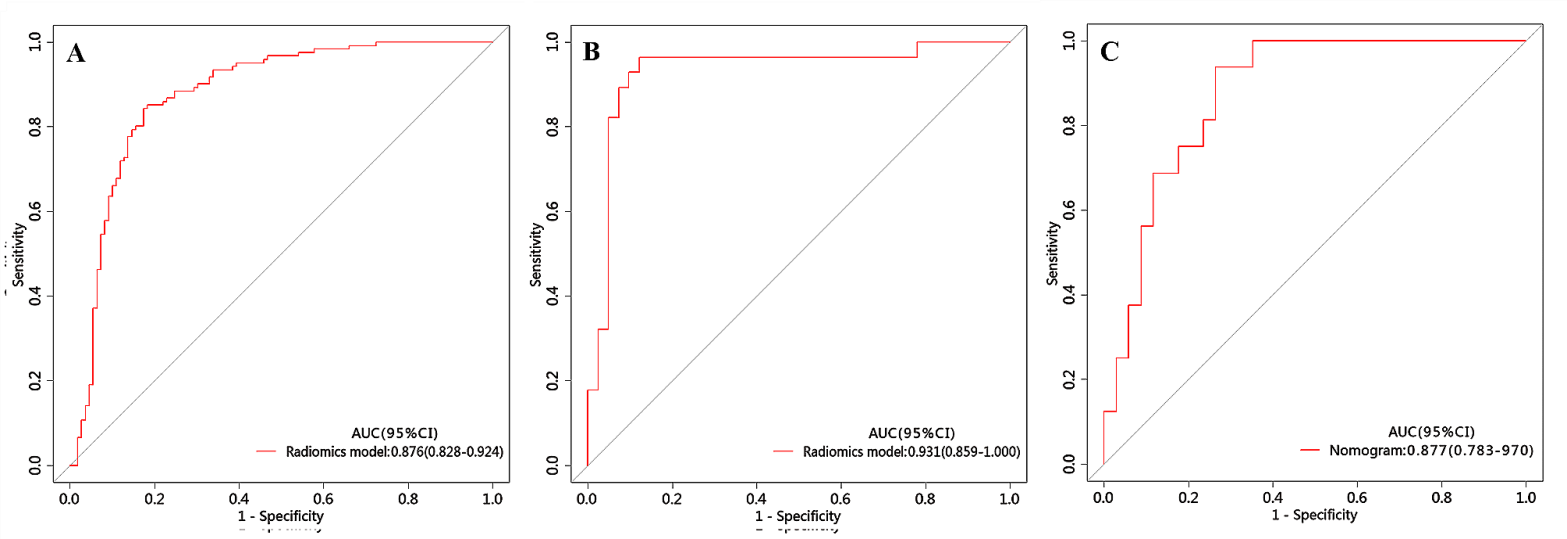
ROC of the radiomics model in the training, internal validation and external testing cohorts

**Fig. 5:**
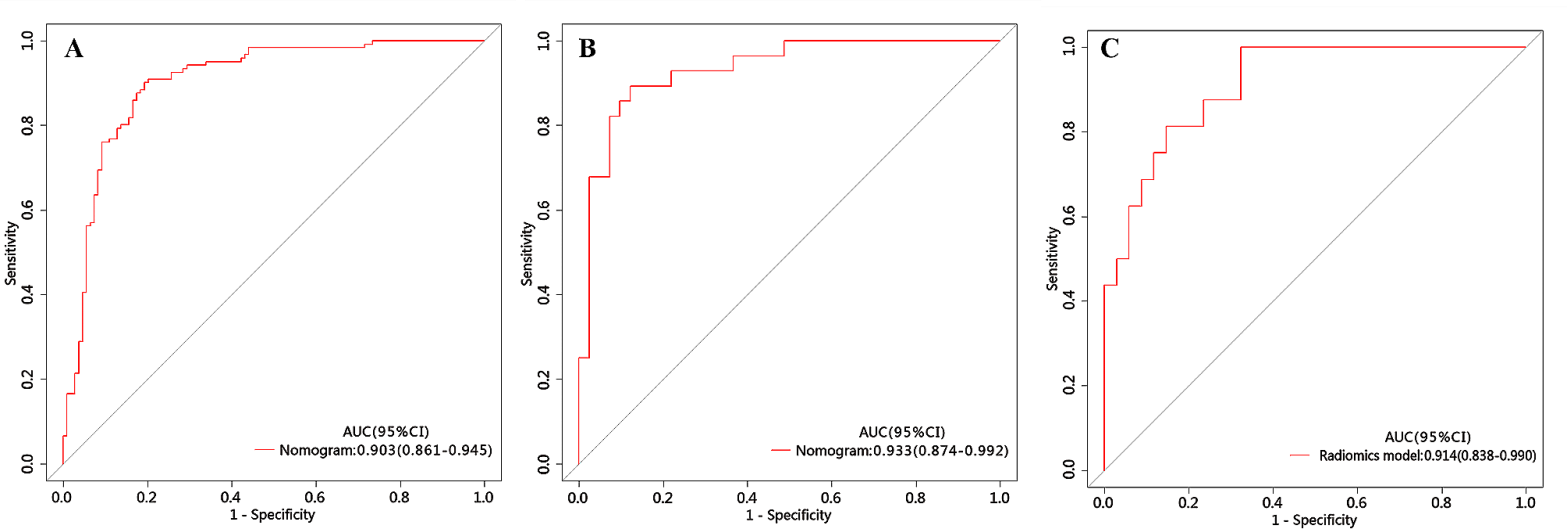
ROC of the nomogram in the training, internal validation and external testing cohorts

The ROC curve was employed to assess the performance of different predictive models in the training, internal validation, and external testing cohorts. The area under the curve (AUC), sensitivity, specificity, and accuracy were calculated. All results pertaining to diagnostic efficacy are presented in Table 4. The ROC curves are displayed in Figs. 3, 4 and 5.

**Table 4:** Performance of three predictive models

Models	Training cohort					Internal validation cohort t					External test cohort			
	AUC(95%CI)	ACC	SEN	SPE		AUC(95%CI)	ACC	SEN	SPE		AUC(95%CI)	ACC	SEN	SPE
Clinical model	0.804 (0.746–0.862)	0.761	0.736	0.789		0.803 (0.684–0.922)	0.826	0.750	0.881		0.597 (0.432–0.763)	0.560	0.812	0.441
Radiomics model	0.876 (0.828–0.924)	0.835	0.843	0.826		0.931 (0.859-1.000)	0.913	0.964	0.878		0.877 (0.783–0.970)	0.780	0.810	0.776
Nomogram model	0.903 (0.861–0.945)	0.857	0.901	0.807		0.933 (0.874–0.992)	0.884	0.893	0.892		0.914 (0.838–0.990)	0.800	0.937	0.835

*Note AUC*, Area under the curve; *CI*, Confidence interval; *ACC*, Accuracy; *SEN*, Sensitivity; *SPE*, Specificity

### Clinical prediction model

The clinical prediction model incorporated independent clinic-radiological predictors (enhanced-CT value and CA125). In the training cohort, this model exhibited an area under the curve (AUC) of 0.804 (95% CI 0.746–0.862) with sensitivity, specificity, and accuracy of 0.736, 0.789, and 0.761, respectively. When applied to the internal validation cohort, the model yielded an AUC of 0.803 (95% CI 0.684–0.922) with sensitivity, specificity, and accuracy of 0.750, 0.881, and 0.826, respectively. Upon validation in the external test cohort, the model yielded an AUC of 0.597 (95% CI 0.432–0.763) with sensitivity, specificity, and accuracy of 0.812, 0.441, and 0.560, respectively.

### Radiomics prediction model

The radiomics prediction model was built based on nine significant radiomics features. In the training cohort, this model exhibited an area under the curve (AUC) of 0.876 (95% CI 0.828–0.924) with sensitivity, specificity, and accuracy of 0.843, 0.826, and 0.835, respectively. When applied to the internal validation cohort, the model yielded an AUC of 0.931 (95% CI 0.859-1.000) with sensitivity, specificity, and accuracy of 0.964, 0.878, and 0.913, respectively. Upon validation in the external test cohort, the model yielded an AUC of 0.877 (95% CI 0.783–0.970) with sensitivity, specificity, and accuracy of 0.937, 0.835, and 0.800, respectively.

### Development and validation of the nomogram

**Fig. 6:**
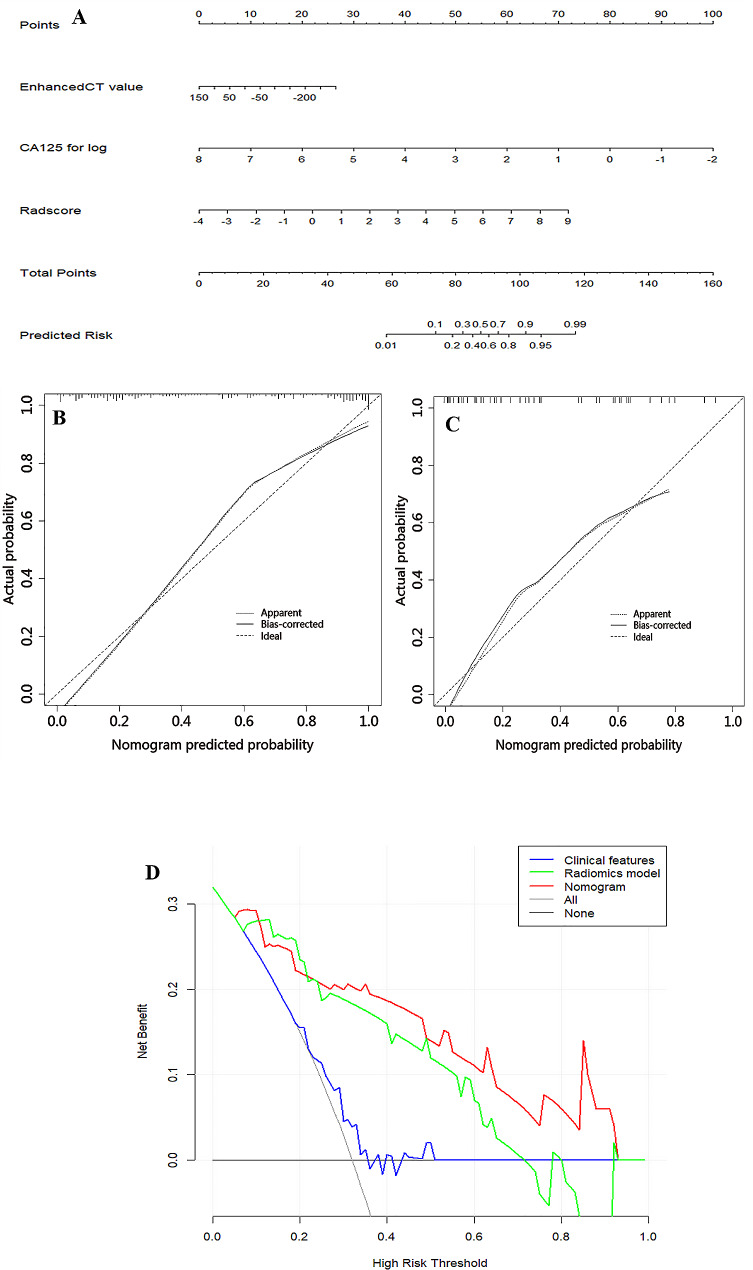
Nomogram for differentiation between tuberculosis granulomas and lung adenocarcinomas based on training cohort and the model evaluation of calibration curve. **(A)** Radiomics nomogram based on clinical characteristics and Radscore. The calibration curves were used to evaluate the consistency between the probability of nomogram prediction and the actual clinical observation in the training **(B)** and external validation **(C)** cohorts. **(D)** DCA for the classification of solitary pulmonary nodules for each model. X-axis represents the threshold probability and Y-axis represents the net benefit. The red curve represents the nomogram. The blue curve represents the clinical model. The red curve represents the radiomics model. The green curve represents the nomogram

**Fig. 7:**
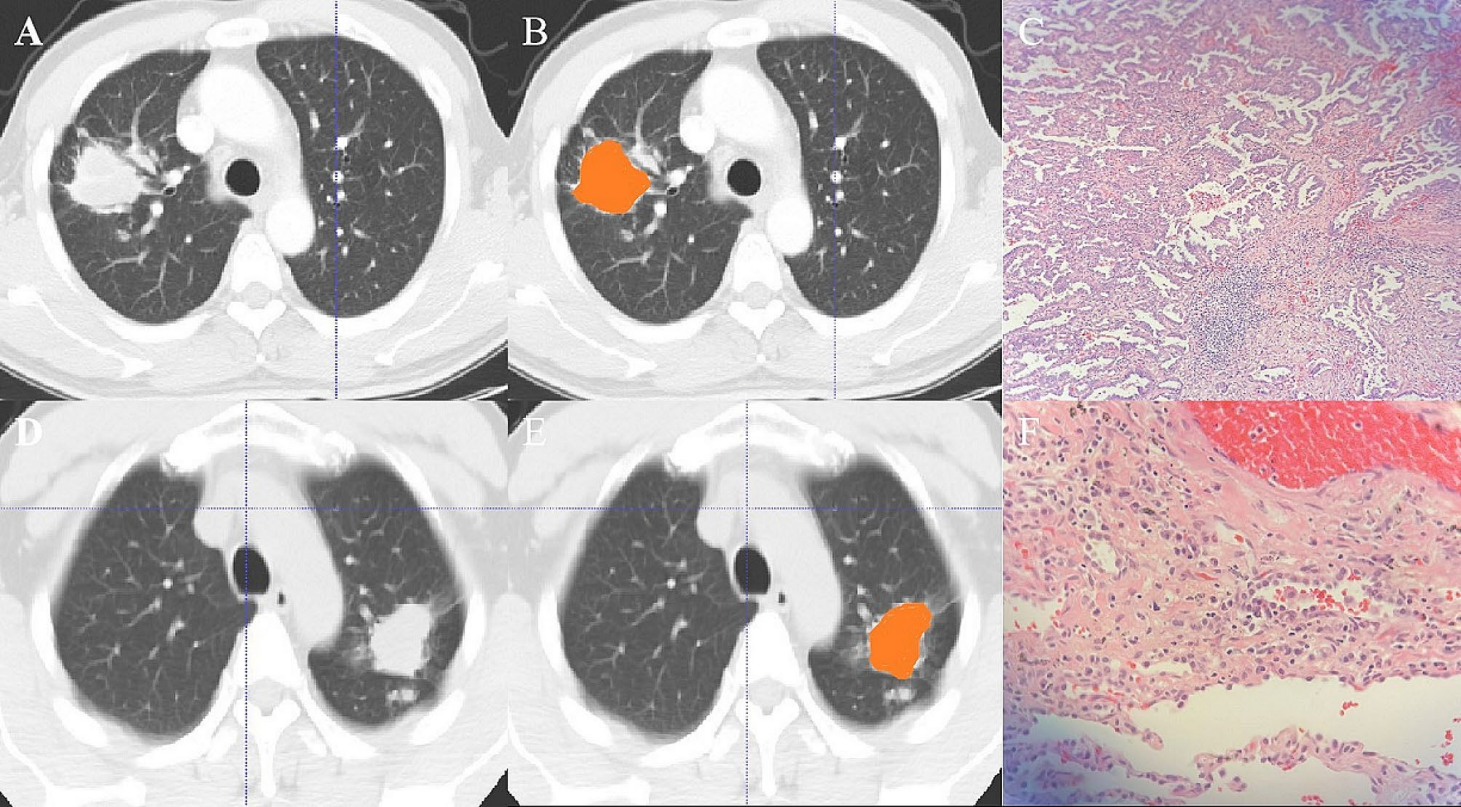
**A-C**: lung window of axial thin-section enhanced chest CT images in a 62-year-old male with proven diagnosis of pulmonary tuberculosis. **(A)** Chest CT image shows, in the right superior lobe, a consolidative opacity. **(B)** The same image showing the consolidative opacity after radiomic volumetric segmentation (in orange). The rad-score calculated by nomogram was 0.76, meanwhile we collected the enhanced-CT value (54.9) and the value of CA125 (19.54). The predicted risk value (0.88) was higher than the cut-off value (0.71), which indicated that a benign lesion. **(C)** The lesion was confirmed on pathological diagnosis as a pulmonary tuberculosis (hematoxylin and eosin, ×400). D-E: In a 58-year-old male, CT scan shows an irregular solid nodule in the left upper lobe. **(D)** Chest CT image shows, in the left superior lobe, a consolidative opacity. **(E)** The same image showing the consolidative opacity after radiomic volumetric segmentation (in orange). We used the same method to obtain the rad-score (0.15), the enhanced-CT value (33.3) and CA125 (8.31). The predicted risk value was 0.62 (< 0.71), indicating the lesion was malignant. **(F)** The lesion was confirmed on pathological diagnosis as a lung adenocarcinoma (hematoxylin and eosin, ×400)

The nomogram is presented in Fig. 6A and satisfactory prediction performance was obtained. In the training cohort, the nomogram exhibited an area under the curve (AUC) of 0.903 (95% CI 0.861–0.945) with sensitivity, specificity, and accuracy of 0.901, 0.807, and 0.857, respectively. Applied in the internal validation cohort, the model yielded AUC of 0.933 (95% CI 0.874–0.992) with sensitivity, specificity, and accuracy of 0.893, 0.892, and 0.884 respectively. Validated in the external test cohort, the model yielded AUC of 0.914 (95% CI 0.838–0.990) with sensitivity, specificity, and accuracy of 0.937, 0.835, and 0.800 respectively. Calibration curves (Fig. 6B, C) indicated that the predicted probabilities of the nomogram were closely aligned with the actual clinical observation in both the training and external testing cohorts. The decision curve of nomogram demonstrated a higher net benefit for the differentiation between lung cancer and tuberculosis than the clinical model and the radiomics model (Fig. 6D). This suggests that the results predicted by our nomogram demonstrated favorable clinical usefulness, and a representative case was displayed in Fig. 7.

### Comparison of different prediction models

**Fig. 8:**
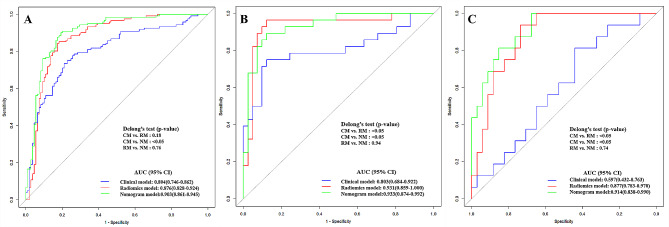
DeLong ROC curves of the three prediction models

Figure 8 demonstrates the ROC curves of clinical model, radiomics model and nomogram in the training cohort, internal validation cohort and external test cohort, respectively. The nomogram demonstrated the optimal discriminative power for pulmonary nodules classification among the three indicators, with improvements in the AUC from 0.804 for the clinical model to 0.903 (*P* < 0.05, DeLong’s test) in the training cohort, from 0.803 for the clinical model to 0.933 (*P* < 0.05, DeLong’s test) in the internal validation cohort, and from 0.597 for the clinical model to 0.914 (*P* < 0.05, DeLong’s test) in the external test cohort. However, the performance of nomogram model did not differ from that of the radiomics model (AUCs 0.903 vs. 0.876; *P* = 0.760) in the training cohort, (AUCs 0.933 vs. 0.931; *P* = 0.940) in the internal validation cohort, (AUCs 0.914 vs. 0.877; *P* = 0.740) in the external test cohort. In contrast, the radiomics model yield a better predictive performance than the clinical model, with improvements in the AUC from 0.803 for the clinical model to 0.931 (*P* < 0.05, DeLong’s test) in the internal validation cohort, from 0.597 for the clinical model to 0.877 (*P* < 0.05, DeLong’s test) in the external test cohort, while the performance of radiomics model did not differ from that of the clinical model (AUCs 0.804 vs. 0.876; *P* = 0.180) in the training cohort.

## Discussion

The current investigation successfully developed a CE-CT radiomics-based nomogram that integrated the radiomics signature and clinical risk indicators to differentiate tuberculosis granulomas from lung adenocarcinomas prior to operation. Our results showed that the enhanced-CT value, CA125 and radiomics signature were significant predictors in differentiating between tuberculosis granulomas and lung adenocarcinomas. The radiomics nomogram is a non-invasive, easy-to-use, personalized approach with excellent performance to preoperatively differentiate tuberculosis granulomas from lung adenocarcinomas, yielding a superior performance with an AUC of 0.903 in the training cohort, 0.933 in the testing cohort, and 0.914 in the external validation cohort, as compared to the clinical or radiomics model alone. This combined radiomics nomogram is conductive to avoid unnecessary surgeries or repeat CT examinations in patients with SPSN.

There is a growing interest in using radiomics for expeditious diagnosis of non-invasive pulmonary CT images among radiologists, which could potentially improve clinical diagnoses. In this study, radiomics features extracted from arterial phase CT images were applied to enhance the differential diagnosis of lung adenocarcinoma and tuberculosis granuloma. The integrated model demonstrated good predictive performance, with a higher predictive ability than the clinical or radiomics model alone. It is noteworthy that the nomogram presented superior diagnostic efficiency and exhibited high prediction ability for the external data. The nomogram is not dependent on the nodules’ conditions, indicating its potential as a non-invasive diagnostic method with a high degree of accuracy for clinicians to obtain results quickly and reliably.

In this study, a clinical model was established that incorporated demographic information and subjective CE-CT findings. By using enhanced-CT value and CA125 as the independent factors, the clinical model achieved a relatively low AUC (0.804 in the training cohort, 0.803 in the internal validation cohort, and 0.597 in the external testing cohort) for differentiating tuberculosis granulomas from lung adenocarcinomas. Therefore, the results confirmed that some CT imaging features did not demonstrate significant correlations with the pathological classification of solid pulmonary nodules. In contrast, the radiomics model yielded better performance in the training cohort (AUC = 0.876), internal validation cohort (AUC = 0.931), and external testing cohort (AUC = 0.877). The radiomics approach can extract large amounts of high-throughput macroscopic features from CT images to quantify lesion information. Thus, the radiomics model enables the extraction of high-dimensional information that is valuable for differentiating tuberculosis granulomas from lung adenocarcinomas in patients with solitary pulmonary nodules compared with the subjective findings model.

The nomogram was developed based on two independent predictors and rad-score. This research explored several significant clinical and radiological characteristics contributing to the differential diagnosis of solitary pulmonary nodules. Univariate analysis showed that six clinical factors (sex, age, mean pack-years, CEA, CA125 and CA199) and three radiological characteristics (spiculated sign, maximum diameter, and enhanced-CT value) were statistically different between the tuberculosis granuloma and lung adenocarcinoma groups. Consistent with the radiologists’ experience, lung adenocarcinoma is more likely to have spiculated sign due to the spread of malignant cells in the pulmonary interstitial. Pathology has demonstrated that spicules’ formation tends to be related to fibrous tissue proliferation or tumor cell infiltration [10]. Previous study has demonstrated that spiculated sign was more likely to be identified as a sign of malignancy on multivariate analysis for screening solitary pulmonary nodules [11]. Although a lobulated shape is often a feature of malignant lesions, it is not an exclusive characteristic, as it can also be observed in benign nodules. The occurrence of a lobulated shape in tuberculosis granuloma was high in our study (119/121). On multivariate analysis, the enhanced-CT value and CA125 remained highly significant in relation to the classification of solitary pulmonary nodules. However, the shape of the lesion (spiculated) was found to only weakly predict the possibility of lung adenocarcinoma. The identification of the spiculated sign on CT images by radiologists was just subjective, and inter-reader variability cannot be ignored. Wang et al. expounded dynamic enhanced CT scanning indicating the value of differentiating lung cancer and pulmonary tuberculosis, in which the enhanced-CT value of adenocarcinoma in the arterial phase is higher than that of tuberculosis (59.27 ± 41.58 vs. 38.88 ± 23.58, *P* < 0.001) [1]. Some studies have shown that the enhancement of tuberculoma was none or mild, and the enhancement peak value were lower than that of lung cancer [12]. This is in agreement with the result of this study. In addition, there was a significant difference in CA125 value between adenocarcinoma and tuberculosis (*P* < 0.001). Specifically, the value of CA125 in adenocarcinoma was generally higher than that in tuberculosis, which was different from the findings of previous study [12]. The increase of the CA125 value in tuberculosis was not prominent, which might be related to the degree or stage of cases enrolled.

In the current study, the 9 core radiomics features were finally retained. Among these radiomics features, Wavelet transform can cover the entire frequency domain and reduce the correlation between different extracted features [13]. The first-order features describe the distribution of voxel intensities in images. The GLCM features quantify the second-order joint probabilities of images which quantifies the intensity distribution of the gray level at a given offset to extract information about tone homogeneity, linear connection, contrast, and boundaries adjacent to gray zones, as well as complicacy of distribution [14]. The GLRLM features describe gray-level runs in an image. Skewness, as one of the simple parameters, represents the asymmetric distribution of gray levels in the histogram that describes the heterogeneity of lesions [15]. Tuberculous granuloma comprises macrophages, including T lymphocytes, B lymphocytes, dendritic cells, fibroblasts, and extracellular matrix components [16]. The above features describe the patterns or spatial distribution of voxel intensities within the ROIs, which serve as recognized parameters to capture tumor heterogeneity [17]. Indirectly, our findings confirmed that the selected features were all closely related to high-dimensional space information that can hardly be understood by naked-eye examination, which may potentially assist in the differential diagnosis.

Several limitations should be considered in this study. Firstly, this study was a retrospective analysis, and selection bias was inevitable. Secondly, the strict inclusion and exclusion criteria resulted in a limited sample size for this study, we did not include GGO nodules. In the future, we will try to include GGO lesions in subsequent studies. Thirdly, this study contained only one external validation, and multi-center collaboration is imperative for us to collect more data to improve the prediction capacity. Fourthly, due to technical limitations, although the intra-nodular radiomics features on Lung CT Images were not extracted, the most important features representing the characteristics of lesions were analyzed.

## Conclusions

In summary, the developed nomogram, which integrates clinical risk factors and radiomics features, demonstrates robust classification performance prior to surgery. Its visualization and interpretability suggest that it has the potential to serve as a valuable and user-friendly tool for personalized decision-making in clinical treatment strategy management.

### Electronic supplementary material

Below is the link to the electronic supplementary material.

Supplementary Material 1

## Data Availability

No datasets were generated or analysed during the current study.

## Abbreviations

ROC: Receiver Operating Characteristics
AUC: Area Under the Curve
CE-CT: Contrast-Enhanced Compute Tomography
ICCs: Intra- and Inter-Class Correlation coefficients
VOI: Volume of Interest
LASSO: Least Absolute Shrinkage and Selection Operator
DCA: Decision Curve Analysis
CI: Confidence Interval
OR: Odds Ratio
