# Supplementary material for: Development and validation of a preoperative CT‑based radiomics nomogram to differentiate tuberculosis granulomas from lung adenocarcinomas: an external validation study
Source: BMC Cancer. 2024 Jun 1;24:670. doi: 10.1186/s12885-024-12422-3 (PMC11144314; doi:10.1186/s12885-024-12422-3)
Supplement: Supplementary file 1 — Supplementary Material 1 [file 12885_2024_12422_MOESM1_ESM.docx]

**Supplemental Materials**

**Supplementary data 1**

**Evaluation of subjective CT findings**

Two experienced radiologists from the“blinded 1” who has 10-year practicing experience in chest disease diagnosis, blinded to the pathological results, independently evaluated the CT images. Specific lung window width (1500 HU window width; −500 HU window level) and mediastinal window width (350 HU window width; 50 HU window level) were used for the analysis of tumor size, location, cavity, vacuole, spicule, satellite lesions, calcification, lobulation, pleural indentation and air bronchogram in CT images. Any discrepancy was settled through consensus by discussion.

**Supplementary data 2**

This section contains the definitions of the various features that can be extracted using PyRadiomics. They are subdivided into the following classes:

• First Order Statistics (19 features)

• Shape-based (16 features)

• Gray Level Cooccurence Matrix (24 features)

• Gray Level Run Length Matrix (16 features)

• Gray Level Size Zone Matrix (16 features)

• Neigbouring Gray Tone Difference Matrix (5 features)

• Gray Level Dependence Matrix (14 features)

Specific radiomic features are listed as follows:

• First Order Statistics (19 features)

1. Energy

2. Total Energy

3. Entropy

4. Minimum

5. 10th percentile

6. 90th percentile

7. Maximum

8. Mean

9. Median

10. Interquartile Range

11. Range

12. Mean Absolute Deviation (MAD)

13. Robust Mean Absolute Deviation (rMAD)

14. Root Mean Squared (RMS)

15. AbsoluteDeviation

16. Skewness

17. Kurtosis

18. Variance

19. Uniformity

• Shape-based (14 features)

1. Flatness
2. Least Axis Length
3. Major Axis Length
4. Maximum 2D DiameterColumn
5. Maximum 2D DiameterRow
6. Maximum 2D DiameterSlice
7. Maximum 3D Diameter
8. MeshVolume
9. Minor Axis Length
10. Sphericity
11. SurfaceArea
12. Surface Volume Ratio
13. Voxel Volume
14. Elongation

• Gray Level Cooccurence Matrix (24 features)

1. Autocorrelation
2. joint Average
3. Cluster Prominence
4. Cluster Shade
5. Cluster Tendency
6. Contrast
7. Correlation
8. Difference Entropy
9. Difference Variance
10. Difference Average
11. Joint Energy
12. Joint Entropy
13. Informational Measure of Correlation (IMC) 1
14. Informational Measure of Correlation (IMC) 2
15. Inverse Difference Moment (IDM
16. Maximal Correlation Coefficient (MCC
17. Inverse Difference Moment Normalized (IDMN)
18. Inverse Difference (ID)
19. Inverse Difference Normalized (IDN)
20. Inverse Variance
21. Maximum Probability
22. Sum Average
23. Sum Entropy
24. Sum of Squares

• Gray Level Run Length Matrix (16 features)

1. Short Run Emphasis (SRE)
2. Long Run Emphasis (LRE)
3. Gray Level Non-Uniformity (GLN)
4. Gray Level Non-Uniformity Normalized (GLNN)
5. Run Length Non-Uniformity (RLN)
6. Run Length Non-Uniformity Normalized (RLNN)
7. Run Percentage (RP)
8. Gray Level Variance (GLV)
9. Run Variance (RV)
10. Run Entropy (RE)
11. Low Gray Level Run Emphasis (LGLRE)
12. High Gray Level Run Emphasis (HGLRE)
13. Short Run Low Gray Level Emphasis (SRLGLE)
14. Short Run High Gray Level Emphasis (SRHGLE)
15. Long Run Low Gray Level Emphasis (LRLGLE)
16. Long Run High Gray Level Emphasis (LRHGLE)

• Gray Level Size Zone Matrix (16 features)

1. Small Area Emphasis (SAE)
2. Large Area Emphasis (LAE)
3. Gray Level Non-Uniformity (GLN)
4. Gray Level Non-Uniformity Normalized (GLNN)
5. Size-Zone Non-Uniformity (SZN)
6. Size-Zone Non-Uniformity Normalized (SZNN)
7. Zone Percentage (ZP
8. Gray Level Variance (GLV)
9. Zone Variance (ZV)
10. Zone Entropy (ZE)
11. Low Gray Level Zone Emphasis (LGLZE)
12. High Gray Level Zone Emphasis (HGLZE)
13. Small Area Low Gray Level Emphasis (SALGLE)
14. Small Area High Gray Level Emphasis (SAHGLE)
15. Large Area Low Gray Level Emphasis (LALGLE)
16. Large Area High Gray Level Emphasis (LAHGLE)

• Neigbouring Gray Tone Difference Matrix (5 features)

1. Busyness
2. Coarseness
3. Complexity
4. Contrast
5. Strength

• Gray Level Dependence Matrix (14 features)

1. Small Dependence Emphasis (SDE)
2. . Large Dependence Emphasis (LDE)
3. Gray Level Non-Uniformity (GLN)
4. Dependence Non-Uniformity (DN)
5. Dependence Non-Uniformity Normalized (DNN)
6. Gray Level Variance (GLV
7. Dependence Variance (DV)
8. Dependence Entropy (DE)
9. Low Gray Level Emphasis (LGLE)
10. High Gray Level Emphasis (HGLE)
11. Small Dependence Low Gray Level Emphasis (SDLGLE)
12. Small Dependence High Gray Level Emphasis (SDHGLE)
13. Large Dependence Low Gray Level Emphasis (LDLGLE)
14. Large Dependence High Gray Level Emphasis (LDHGLE)
